# 3D printing-based frugal manufacturing of glass pipettes for minimally invasive delivery of therapeutics to the brain

**DOI:** 10.1002/nep3.20

**Published:** 2023-06-19

**Authors:** Guanda Qiao, David Gulisashvili, Anna Jablonska, Guiling Zhao, Miroslaw Janowski, Piotr Walczak, Yajie Liang

**Affiliations:** 1 Department of Diagnostic Radiology and Nuclear Medicine, University of Maryland School of Medicine, Baltimore, Maryland, USA; 2 Laboratory of Molecular Cardiology, Department of Physiology, Center for Biomedical Engineering and Technology, University of Maryland School of Medicine, Baltimore, Maryland, USA

**Keywords:** 3D-printable, brain injection, frugal science, glass pipette pulling

## Abstract

**Objective:**

Intracerebral delivery of agents in liquid form is usually achieved through commercially available and durable metal needles. However, their size and texture may contribute to mechanical brain damage. Glass pipettes with a thin tip may significantly reduce injection-associated brain damage but require access to prohibitively expensive programmable pipette pullers. This study is to remove the economic barrier to the application of minimally invasive delivery of therapeutics to the brain, such as chemical compounds, viral vectors, and cells.

**Methods:**

We took advantage of the rapid development of free educational online resources and emerging low-cost 3D printers by designing an affordable pipette puller (APP) to remove the cost obstacle.

**Results:**

We showed that our APP could produce glass pipettes with a sharp tip opening down to 20 μm or less, which is sufficiently thin for the delivery of therapeutics into the brain. A pipeline from pipette pulling to brain injection using low-cost and open-source equipment was established to facilitate the application of the APP.

**Conclusion:**

In the spirit of frugal science, our device may democratize glass pipette-puling and substantially promote the application of minimally invasive and precisely controlled delivery of therapeutics to the brain for finding more effective therapies of brain diseases.

## 1 | INTRODUCTION

Multiple routes are used for administering therapeutics (chemical compounds, gene vectors, or cells) to the brain, such as intraperitoneal, vascular-based (intravenous or intraarterial),^1,2^ intraventricular,^3,4^ intranasal,^5^ and intracerebral injections, with their advantages and disadvantages. Among these routes, the intracerebral injection is characterized by precise targeting, direct access to brain parenchyma bypassing the blood–brain barrier, and ease of operation.^6–8^ However, the main disadvantage is its invasive nature and tissue damage due to the relatively large size and rough surface of commercially available, durable metal needles. One way to minimize the harm associated with direct intracerebral injection is to use tools with thinner tips and smoother surfaces than these needles. For example, because of their excellent extension properties, glass pipettes can be pulled to form a tool with a much thinner internal diameter and wall thickness than metal needles for minimally invasive brain injection of drugs, gene vectors, or cells.^9–11^

Glass pipettes are widely used in life sciences for a wide range of applications. Traditionally, one main application in neuroscience is electrophysiology as electrodes for intracellular recording,^12^ patching,^13^ or delivery of minute volumes of materials (DNA or dyes) to cells.^14^ However, in recent years, there has been a sharp increase in the use of glass pipettes for the delivery of adeno associate virus (AAV) into the brain, partly because of the booming of genetically encoded indicators or actuators, such as calcium indicators or optogenetic tools and convenient access to optical imaging systems for recording neuronal activities. Glass pipettes are also used to inject cells into the brain for therapeutic purposes,^15,16^ although the majority of the literature utilizes Hamilton syringes connected to metal needles as delivery tools.^17–21^

Despite the many advantages of delivering vectors or cells into the brain using pulled thin-tip glass pipettes, especially for preclinical studies, a major hurdle for their widespread application is accessibility and affordability. For electrophysiological labs already equipped with programmable pullers, it is handy to repurpose them to make glass pipettes^22^ for delivering fluids, such as AAV into the brain. However, for nonelectrophysiological labs, in which such programmable pipette pullers are not essential for daily work, these pricey programmable pullers create an economic barrier for many labs to obtain correctly pulled glass pipettes. Furthermore, even for well-funded nonelectrophysiology labs, it seems an overkill to spend thousands of dollars purchasing a professional programmable pipette puller just for making viral-injection level glass pipettes, which require much less precision than those for patching experiments.

To avoid using over-kill techniques and to properly allocate scientific resources, we designed an open-source 3D printable device to pull glass pipettes along with a platform to prepare glass pipettes ready for the delivery of therapeutics into the brain (mouse brain in our case). The cells can also be delivered using glass pipettes processed using our platform. Our system mitigates the economic burden of glass pipette pulling and makes the glass pipette-based delivery system accessible to more laboratories.

## 2 | METHODS

### 2.1 | Design and 3D print affordable pipette puller (APP) using open-source software

The APP was designed in Tinkercad and exported as individual. stl files. Then, individual parts were sliced in Ultimaker Cura at 0.2 mm precision, 60% infill, and saved as gcode files. CREALITY Ender 3 v2 (Shenzhen Creality 3D Technology Co.) was used for 3D printing with 1.75 mm diameter polylactic acid filament. Each 3D-printable item was printed at 50% of speed. Nozzle temperature was 200°C. Bed temperature is 50°C. A total of 0.4 mm diameter nozzle was used. Glass capillaries have the following dimensions: outer diameter (OD): 1 mm; inner diameter (ID): 0.78 mm, purchased from Warner Instruments (G100T-4,# 640778). Design files can be found here: https://github.com/liangy10/APP_platform.

### 2.2 | Assembly of the APP and using it for pulling glass capillary

The stands (×2), bases (×2), and sliding pipette holders (×2, one has a size wall) were 3D printed and assembled with a 4 mm diameter metal rod (20 mm in length, see bill of materials [BOM], in Supporting Information). Rubber bands (see BOM) were selected to provide the desired force measured by a digital force gage (BAOSHISHAN, China #ZP-500N, https://www.amazon.com/BAOSHISHAN-Rechargeable-Battery%EF%BC%88Kgf-Dynamometer-Destructive/dp/B082KNR997?th=1). A glass capillary (1 mm ID, 10 μL volume, #5–000-2010, Drummond) was fixed to the central canal of pipette holders fastened by the pipette cover using M4 screws. A hand-held butane burner (see BOM) was positioned right beneath the pipette through the adapters and fired till the glass pipettes were pulled apart. For the beveling of the pipette tip, a used external hard drive (Newman Star, USB2.0) was repurposed. A sandpaper (1500 grit, see BOM) was cut and glued on top of the disc of the hard drive.

### 2.3 | Measurement of tip size for pulled glass pipettes

A low-cost microscope (YEGREN, see BOM) was used to visualize the tip of the pulled glass pipettes using 4× or 10× objectives. An image of the microscope camera calibration slide (OMAX, see BOM) was taken by a USB camera using Snap Camera (https://snapcamera.snapchat.com/). The scale was set in the image using ImageJ to measure the tip size of glass pipette images under the same objective. The microscope reticle was made based on the instructions in this video: https://www.youtube.com/watch?v=uDsqgjPK3b8. Briefly, a 20 μm-long mark was made on a transparent thin plastic disc which was then put on the plane of focus for the eyepiece. The mark will be superimposed on the image observed through an eyepiece or USB camera.

### 2.4 | Animal, stereotaxic procedures, and histology

This study was performed in accordance with the National Institutes of Health Guide for Care and Use of Laboratory Animals and the University of Maryland, School of Medicine, Animal Care and Use Committee. One male C57BL6J mouse was used for in vivo testing. One microliter of green fluorescent beads (505/515, 0.1 μm diameter, Thermofisher, #F8803, 25 times diluted with phosphate buffer solution (PBS) was injected into the cerebral cortex using either pulled glass pipettes (right side of the hemisphere) connected to a Narishige micromanipulator (#MO10) or Hamilton syringe needle 33 Gauge (left side of the brain) connected to a motorized pump (KD Scientific, #78–8130). The Bregma coordinates for the three injection sites for each hemisphere were −1, 0, and 1 mm. Midline and depth were the same among the sites: 2 and 0.8 mm, respectively. The injection speed was 0.5 μL/min. The animal was perfused with 4% paraformaldehyde 11 days after the injection of fluorescent beads and dehydrated in 30% sucrose in PBS before being cryosectioned (Cryostar NX50, Epredia) at 40 μm for observation under fluorescence microscopy (Leica).

### 2.5 | Statistics

All statistical values are presented as mean ± standard deviation unless otherwise noted. Box-and-whisker plots were used in Figure 2 center line, median; box limits, upper and lower quartiles; whiskers, 1.5 × interquartile range.

## 3 | RESULTS

**FIGURE 1:**
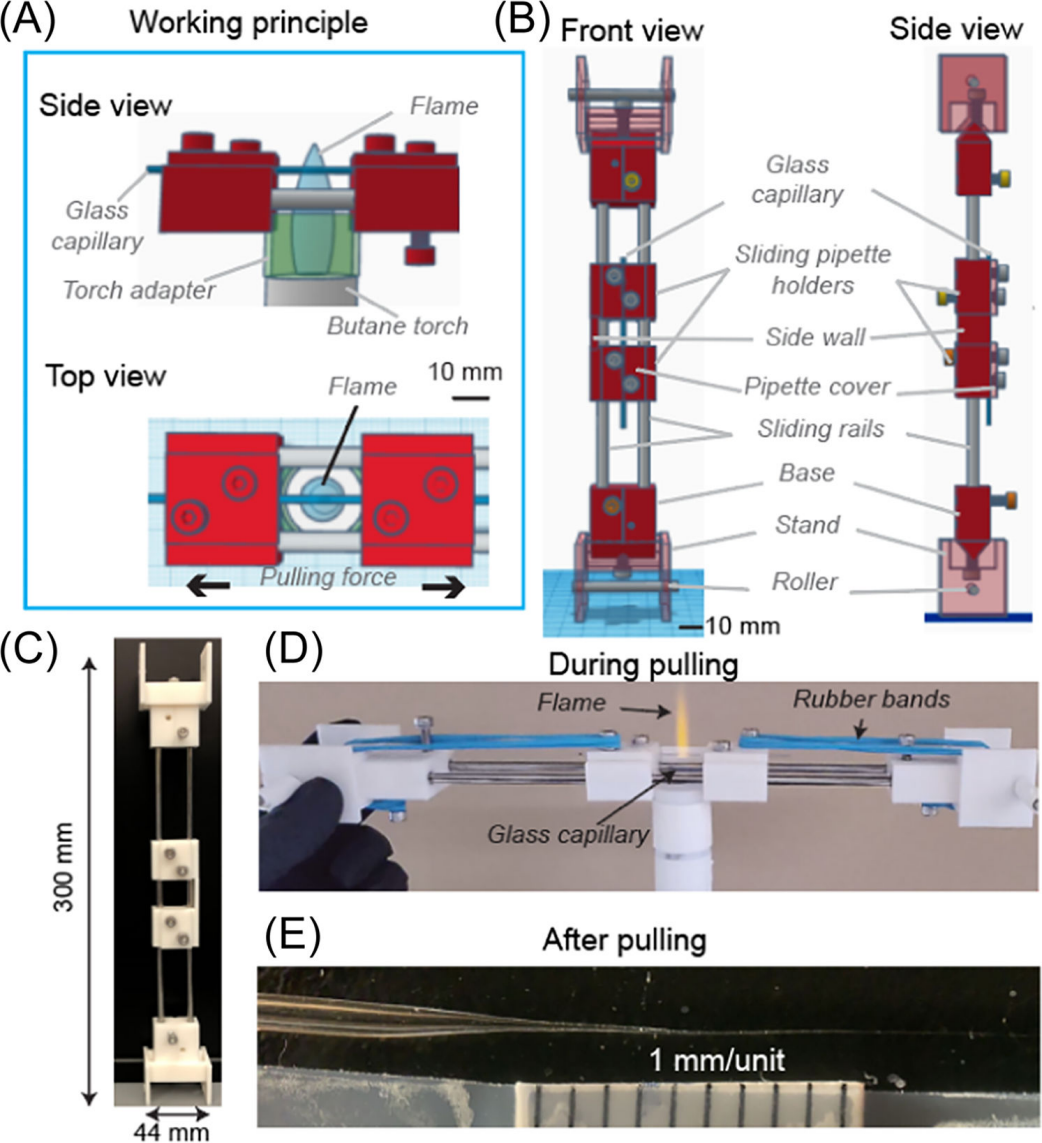
Design of the affordable pipette puller (APP). (A) Working principle of APP. Side and top view of the APP in action. (B) The design and components from our APP. (C) Assembled APP with the dimension of 300 (height) × 44 (width) × 30 (length). (D) Pulling procedure. (E) After pulling, a thin pipette tip was formed with a long neck. Scale bar is 10 mm for all panels.

Like the programmable pipette puller,^23^ we first sought an accessible heat source that is hot enough to melt the glass quickly. The butane torch is an affordable device to produce high temperature (1200–1400°C)^24,25^ to melt glasses, which is also easily accessible from hardware stores. Therefore, it can be an affordable heating source to pull the glass capillary apart. The working principle of APP is illustrated in Figure 1A. The glass capillary was sandwiched between 3D-printed pipette holders, and pipette covers were tightened by two pairs of M4 screws. A hand-held butane blow torch was positioned below the glass capillary between the two pipette holders and isolated by the *Torch adapter*. A pulling force was applied from both ends of the glass pipettes while the torch was switched on until the glass pipette was split into two. High-quality rubber bands are anchored to the bases at one end and sliding pipette holders at the other end, providing a strong pulling force (in Newtons, N). We used a pair of metal rods as the sliding rail to allow the broken glass pipette to move smoothly away from the head source. Mineral oil was used as a lubricant. The APP was designed using Tinkercad, an open-source online software consisting of 3D printable and off-the-shelf parts (Figure 1B). Two *sliding pipette holders* fix the pipette at a distance that allows the insertion of a blow torch in between. This distance is controlled by the length of the *side wall*, which should be customized depending on the size of the flame source. The *Bases* of the APP serve as anchors for rubber bands and stop the movement of *sliding pipette holders* at the end of the pulling procedure. There are two stands at the end of the APP for proper positioning in the laboratory space when it is not in use. The *roller* in the *stand* facilitates the transfer of the rubber band force throughout its full length for an accurate evaluation of the pulling force. After the assembly of the APP (Figure 1C), rubber bands were used to provide pulling force under a hand-held butane flame (Figure 1D). The whole pulling procedure was brief (a few seconds), generating a long taper with a very thin tip (Figure 1E). A slow-motion video of the entire pipette-pulling process can be found in Supporting Information (Movie 1). Depending on the pulling parameters, the tip could be so thin (a few micrometers in diameter) that it was free-floating in air. It indicates that the glass pipettes generated using our APPs are thin enough (<20 μm in diameter) for the delivery of therapeutics in the brain, even though they may not be suitable for cell patching, which is more demanding in terms of the shape, size, or length of the shaft.

**FIGURE 2:**
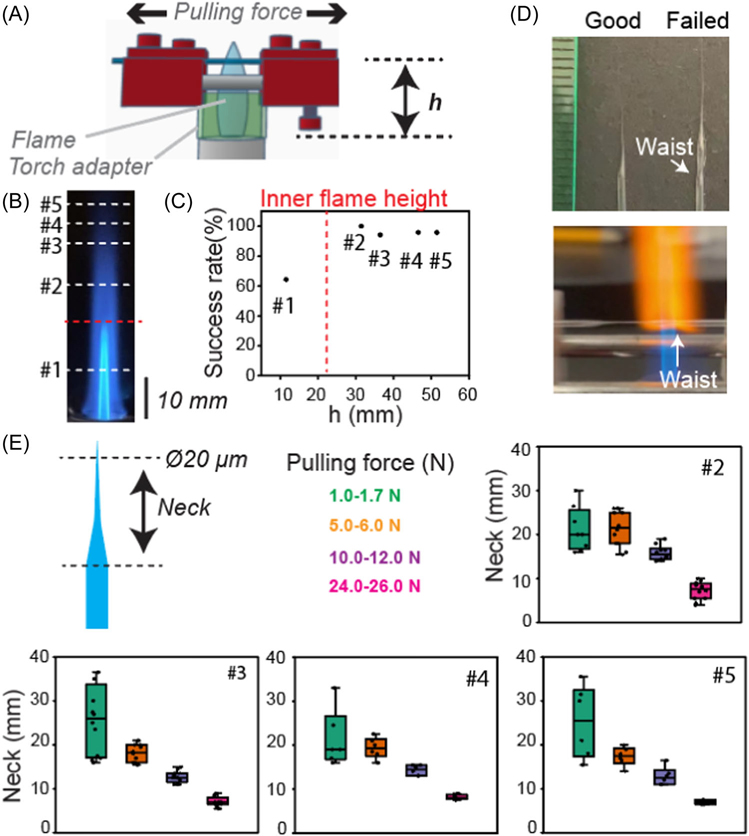
Characterization of essential parameters for pipette pulling using the affordable pipette puller (APP). (A) The pulling force and flame height are two critical variables. (B) The flames of the hand-held butane torch and positions tested. (C) Success rate as a function of the flame height. The red line indicates the height of inner flame in (B, C). (D) Side-by-side comparison of good and failed pipettes and a slow-motion image showing waist formation during the pulling process. 1 mm/unit of length. (E) The definition of the length of the neck and neck distance under different pulling forces (1.0–1.7 N, 5.0–6.0 N, 10.0–12.0 N, 24.0–26.0 N) and flame heights (#2 through #5).

Next, we characterized the variables essential for successfully producing usable glass pipettes using our APP. The most important variables were the height of the flame and the horizontal pulling force. The height of the flame was defined as the distance from the base of the flame to the glass pipette at the pulling position (*h*, Figure 2A). We tested five positions (#1 through #5) and found that when ***h*** was too small (as in position #1, Figure 2B), there was a high failure rate with failure defined as at least one of the pulled pipettes not being usable. However, as ***h*** increases, the success rate improves and is maintained at a certain level (Figure 2C). Thus, to ensure a high success rate, we recommend keeping the glass pipette above the inner flame. We then took a closer look at this failed pipette (Figure 2D, upper panel), which is not usable because of the presence of a *waist*, defined as a narrowing of the glass pipette close to the neck. To determine what happened, we recorded a video of slow motion during the pulling process (see Movie 2 in Supporting Information) and found that the waist is another potential breaking point on the opposite side of the inner flame, away from the first breaking point during the heating process (Figure 2D, lower panel). Regarding the pulling force (in N), we first defined the neck length as the distance from the converging edge to the pipette tip with a diameter of 20 μm (Figure 2E, upper left panel). Because all the pulled glass pipettes had an opening of <20 μm, we normalized the neck length by cutting off the tip part with a diameter of <20 μm. As shown in Figure 2E across different flame heights, the higher the pulling force, the shorter the neck. Another apparent trend is that the jitter within the group also decreased as the pulling force increased. Therefore, pulling forces should be adjusted accordingly depending on the neck length needed for a particular application; however, a higher force may produce more consistent results.

To gain insight into the difference between our APP and programmable pipette pullers, we pulled the same type of glass pipettes with a widely used programmable puller Sutter Instrument P-97 for electrophysiology labs. We noticed two main differences. First, the neck is much shorter when using P-97 (Supporting Information: Figure 1A,B). We reason that this is due to the lack of delay after heating, which was supported by the graph showing that pipette necks became longer as the delay time was reduced (Supporting Information: Figure 1C). This indicates that another way to reduce the neck length (other than the stronger pulling force mentioned in Figure 2) is to have delay time after heating, which requires precise control of the timing during the pulling procedure. The second main difference between our APP and programmable pipette pullers is the sharp tip (<1 μm) for P-97 pulled pipettes (Supporting Information: Figure 1D), which cannot be achieved through APP. To test the durability of our APP, we continuously pulled 25 times, each time using a new glass pipette. Forty-nine glass pipettes were obtained with one broken during the handling procedure (not during pulling, Supporting Information: Figure 1E). Then, we measured their neck distance and found high consistency as pulling numbers increased (Supporting Information: Figure 1F). This is strong evidence to support the long-term durability and reliability of APP for glass pipette pulling.

**FIGURE 3:**
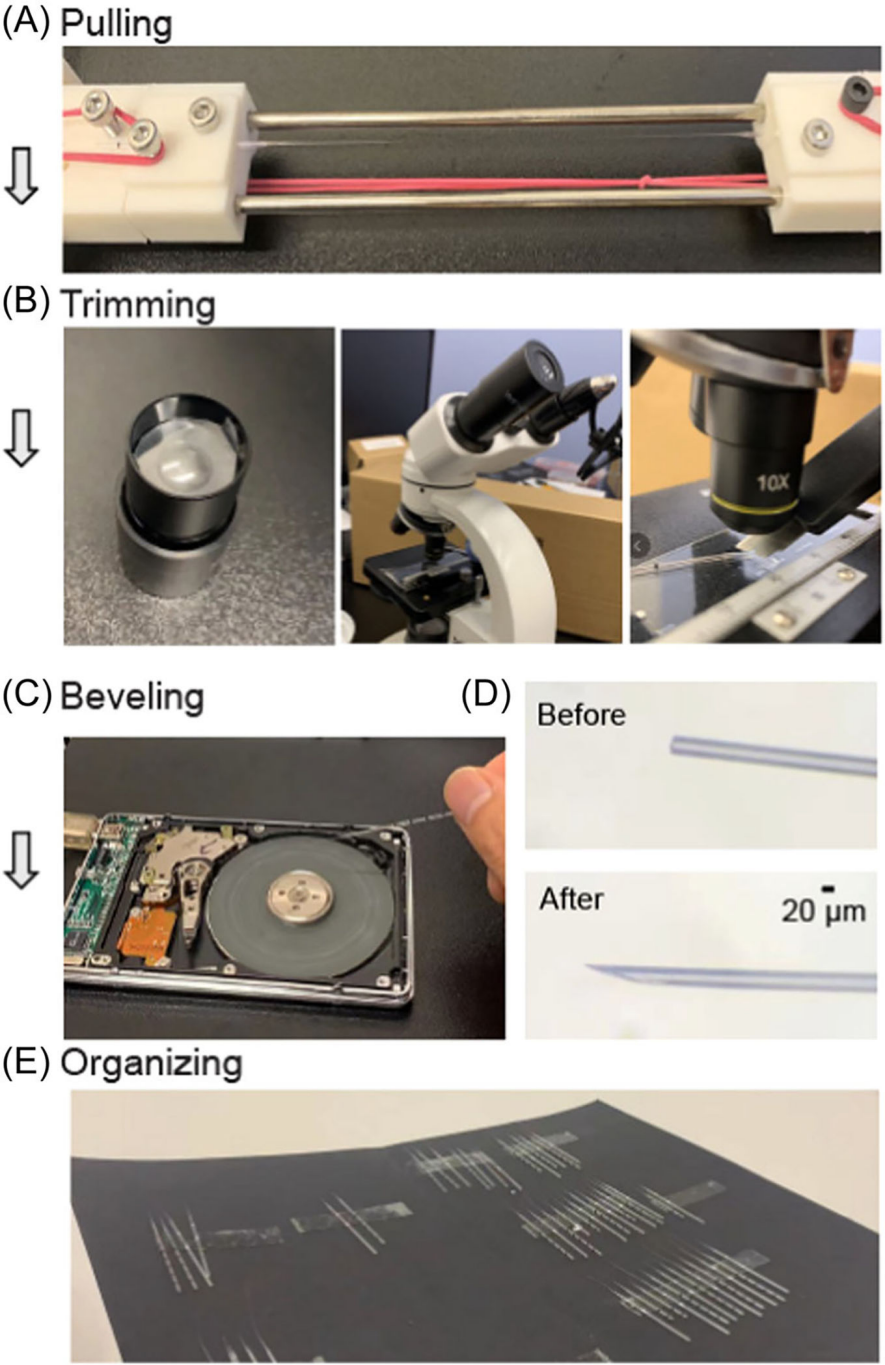
The platform of making ready-to-use pulled glass pipettes. (A) Pipette pulling using affordable pipette pullers (APPs). (B) Trimming the pulled glass pipettes to the desired diameter at their opening with the help of a reticle. (C) Beveling the tip of the glass pipettes with a used hard drive covered with sandpaper. (D) Comparison of before and after the beveling process. (E) Pipettes are organized by gluing them on hard paper. Scale bar is 20 μm.

Going downstream, we established a platform for processing capillaries ready to inject therapeutics. After pulling the glass pipettes (Figure 3A), we trimmed the tip to the desired diameter (Figure 3B), for example, 20 μm, which is thin enough for liquid delivery into the brain, or 40–50 μm for cell delivery. A low-cost bright-field microscope was used to visualize the tip at 100× magnification. The microscope was placed with an eyepiece reticle with the desired length (20 μm, e.g., Figure 3B, left panel), which was used to center the glass pipette at the correct position. Then, a small flashlight was inserted into the eyepiece to illuminate the part of the glass pipette to be removed (Figure 3B, middle panel) by cutting it with a knife (Figure 3B, right panel). It generated a glass pipette with a tip opening diameter of the desired size. Next, we currently have a blunt or irregular ending for the pipette tips, which is unsuitable for brain penetration. Therefore, they need to be beveled. Borrowing from early literature,^26^ we adapted a used external USB hard drive for this purpose (Figure 3C). The hard drive cover was retained as a dust shield. The hard drive was covered with 1500 grit sandpapers for beveling 20 μm diameter pipette tips. 800-grit sandpaper could be used for beveling glass pipettes with larger openings. After a few seconds of beveling at sharp angles, the glass pipette was examined under a microscope. If performed correctly, this procedure will yield good results, as evidenced by the appearance of a well-beveled ending (Figure 3D). If large-scale pipette pulling is required, we recommend organizing them on foldable hard papers using double-sided glue to align them (Figure 3E).

**FIGURE 4:**
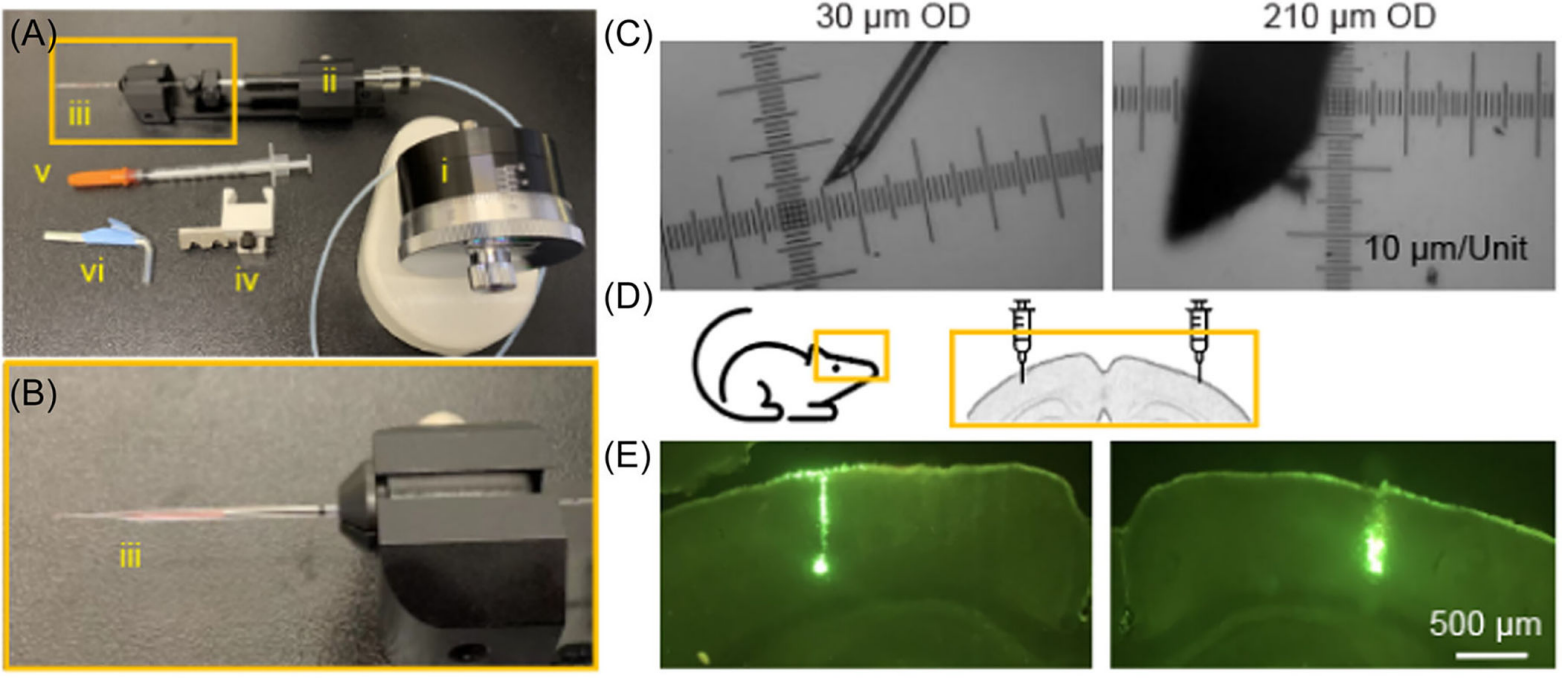
Injection of fluids into the mouse brain through glass pipettes or a Hamilton syringe needle. (A) Components of the injection system. (i) micromanipulator; (ii) pipette holder; (iii) pulled glass pipette filled with mineral oil (0.1% Sudan red stained); (iv) adapter to the stereotactic arm; (v) syringe needle to load the mineral oil; (vi) hex key for the pipette holder; (B) Zoom-in of part (iii). (C) The glass pipette (left) or Hamilton 33 G needle (right) used for brain injection. A total of 10 μm for the smallest unit. (D) Schematic of the experimental design. (E) Representative histology images showing the needle track made by glass pipettes (left) or Hamilton needle (right). Scale bar is 500 μm.

After cleaning or autoclaving if needed, the pulled glass pipettes are ready to be assembled with nanoliter-scale injectors for the delivery of fluid at high precision (Figure 4A). We used the stereotaxic injector system designed by Scot Sternson lab (Janelia Research Campus, Howard Hughes Medical Institute) which is an open-resource design (https://www.janelia.org/open-science/stereotaxic-injector-system). The injector system was mounted on a stereotaxic frame (data not shown) for injection and consisted of three parts: an oil hydraulic micromanipulator (Figure 4A item i), a micropipette holder (Figure 4A item ii), and a glass pipette with a sharpened tip (Figure 4A item iii). Additional items included an adapter to the stereotaxic arm (Figure 4A item iv), a syringe with a 29 G needle (Figure 4A item v) for loading mineral oil into the glass pipettes (for AAV injection), and the Hex key to loosen or tighten the hold on glass pipettes (Figure 4B). Under our settings, the progression of 50 μm in the hydraulic micromanipulator results in the dispensing of 10 nL of solution from the glass pipette, that is, 0.2 nL/μm progression, enabling high-precision fluid delivery at the nanoliter scale level. We then compared a 30 μm OD glass pipette (Figure 4C, left panel) with a Hamilton syringe needle of size 33 gauge (Figure 4C, right panel) for injecting 1 μL of fluorescent beads (0.1 μm diameter) into the mouse brain (Figure 4D). Histological analysis revealed a narrower track in the glass pipette-mediated injection than that in the 33 gauge needle (Figure 4E). This indicates less brain damage associated with injections mediated by glass pipettes due to the smaller diameter (30 vs. 210 μm). Thus, our in vivo testing confirmed the advantage of using pulled glass pipettes with thin tips over conventional metal-based syringe needles.

## 4 | DISCUSSION

In this study, we designed an open-source 3D printable device, APP, to create glass pipettes with sharp ends up to 20 μm ID or less, which is sufficient for the minimally invasive delivery of fluids into the brain. Larger IDs are needed for the delivery of cells, which falls within the engineering range of our APP. Our design eliminates the need to purchase prohibitively-expensive programmable pipette pullers (a few thousand to ten thousand dollars) and reduces the cost of pipette-pulling devices to <10 dollars. We were inspired by the concept of frugal science,^27^ which utilizes curiosity-driven thinking to leverage common everyday items and repurpose them to solve complex engineering problems for bioengineering. Overall, our device democratizes glass pipette pulling and makes this technique more accessible and available to the biomedical community, especially for small labs or labs in less developed countries.

Traditionally, one of the primary uses of glass pipettes is for electrophysiologists to record electric signals from excitable cells by patching the cell membrane, for example. It is very demanding, considering the dimensions of the glass pipette tips. By precisely controlling the heating and pulling, programmable pipette pullers reliably produce glass pipettes of desired dimensions (shaft length, tip diameter, and tip shape) for patch-clamp or intracellular recording. The advantage of using a programmed puller is the precision of the puller process, which enables stable control of the size and shape of the pipettes. These parameters are essential for delicate intracellular or patch-clamping experiments. Therefore, programmable pullers represent a remarkable breakthrough that liberated the glass electrode field from the labor-intensive and craftsmanship-dependent manual pulling of capillaries in the 1920s through a must-have tool in electrophysiological labs.^28^ However, there are no available low-cost options for tasks that are not demanding, such as injecting fluids or cells into the brain. The Sutter Instrument P series (30, 97, 1000, or 2000) or Narishige (pe-22, PC10, PC100) micropipette pullers cost thousands of dollars. It creates an economic barrier for most biomedical research wet labs, which cannot easily access programmable pipette pullers. With the wide application of viral vectors, especially AAV, for studying brain activity, molecular biology, and metabolism and for testing therapeutic use, there is an increasing demand for thin glass pipettes to deliver viral vectors into the brain. Compared with conventional Hamilton syringes, thin glass pipettes have three obvious advantages. First, they cause less damage to the brain owing to their small diameter tip size. Second, they could cover more brain areas if more injection sites are made, enabling more evenly distributed transduction of the target tissue. Third, much less volume is needed when using glass pipettes (<100 nL)^29–31^ compared to 1–2 μL when using Hamilton syringe needles.^19–32^ Therefore, our APP will meet the increasing need for proper tips for delivering AAVs into the brain, breaking the economic barrier caused by the need for expensive devices, and democratizing glass pipette pulling.

Through thorough characterization of the pulling settings, we explored how different variables affected the dimensions of glass pipettes and established their viable ranges for successful pipette pulling. The most important parameters were the height of the flame (distance between the glass pipette and the base of the flame) and the horizontal pulling force exerted on the pipette. We found that if the pipette was heated within the inner flame of the torch, there was a high chance of obtaining only one usable pipette out of the two resulting pipettes. The failed pipettes have a waist close to the neck of the pipettes, which prevents the insertion of the plunger to the very end, and thus can hardly be used. The slow-motion video revealed that the waist is another potential breaking point on the other side of the inner beam, which is likely due to the higher temperature at the rim of the flame than at the center where the inner flame resides, which is consistent with the temperature distribution in a flame.^33,34^ To prevent this from occurring, we recommend avoiding using an inner flame for heating glass pipettes, which yielded a higher success rate (Figure 3C). Regarding the force applied to the pipettes, we found that the higher the force, the shorter the neck (Figure 3E). Therefore, the force can be adjusted depending on the desired length of the neck (or taper) of the pipette. The inner tip diameter for our pipettes was always smaller than 20 μm, which is why we needed to normalize them by having a 20 μm cutoff value for the quantification of neck length. This size is small enough for minimally invasive delivery of AAVs or other fluids into the brain. For the delivery of cells, a larger opening is necessary, such as 30–50 μm, which only requires cutting pipette ends at a higher level to create a larger opening.

We also established a pipeline from pipette pulling to the fluid injection. Pipette pulling is the first step in the delivery of therapeutics into the brain. We created a simple method to quickly trim the pipette to the desired diameter using an eyepiece reticle and highlight the target pipette position with reverse illumination through the eyepiece. The tip of the glass pipette must be beveled for easy penetration of the brain dura. We adopted a convenient, low-cost way^26^ to bevel the glass pipette using a hard drive with a sand sheet glued to the disc. It created a sharp tip in the pulled glass pipettes. The pipette could then be used for brain injection by connecting it to an injector. Nanoject (Drummond) is the most frequently used microinjector. Another option is the manually controlled microinjector developed by Scot Sternson lab^35^ from JRC, HHMI, which allows for more precise control of the amount of liquid for delivery. Our in vivo test confirmed less brain damage associated with brain injections mediated by 30 μm diameter glass pipette compared to that mediated by the 33 G Hamilton needle (Figure 4E). Overall, our platform paves the way for low-cost open-source preparation of glass pipettes and precisely controlled brain injection. The user-friendliness of our APP is important for labs in less developed countries.

Several limitations must be borne in mind for using our APP. First, the low precision of household 3D printers and low-cost items require alignment of the device and adjustment of dimensions in the design. Each printer may have different precision levels, creating variations in the compensation size for parts that need to be matched to each other. For example, steel rods have a diameter of 4 mm which will fit the 4 mm diameter hole in the 3D printed parts owing to the imprecision in 3D printing. Therefore, for sliding rods of 4 mm, we set the holes to be 4.1 mm of diameter in the base and 4.3 mm diameter in the sliding pipette holders. Second, there is a limit to the pulling force, which corresponds to a lower limit of the taper length. It was found that when the pulling force was 30–40 N, there was a risk of structural failure. Thus, the recommended safe pulling force is <30 N, although users may need to figure out this limit in their hands owing to variations in printing and assembling procedures. Third, the pulling force provided by the rubber bands may decay over multiple applications. It is our experience that rubber bands provide the highest force upon their first use, which will continue to decrease in subsequent uses. Therefore, the pulling forces exerted by rubber bands should be actively measured if more precision is required. Fourth, the taper of the pulled pipette may not be straight. For a pipette with a long taper or neck, we noticed that the taper is often crooked, that is, unparallel to the body of the pipette. It may be caused by the misalignment of structures or pulling forces. Although the crooked angle is small and does not affect the application of solution injection, the APP has to be carefully aligned to minimize this problem. Fifth, there are safety concerns. When pulling the pipettes at a high force, there is a risk of minor crush injury if the fingers of the users are between the sliding and fixed pieces in the APPs. Another safety issue concerns the use of open flames. We recommend that any use of our APP should be performed in well-ventilated areas with more than one person. Safety should always be a priority when using any laboratory device. The precision, consistency, and repeatability of the pulling process or results are dependent on the quality control during the manufacturing, assembling, and calibrating procedures. In our hands, the small pulling force (e.g., 1 N in Figure 2) is associated with higher variation of the neck length as shown in Figure 2E. Thus, we recommend pulling at 10–20 N force for best consistency and repeatability.

## Supplementary Material

supplementary figure

supplementary video1

ssupplementary data

supplementary video 2

## Data Availability

All designs are available on GitHub (https://github.com/liangy10/APP_platform). A detailed list of materials (Bill of materials, BOM) used in this study can be found in the Supporting Information.

## Highlights

- Open-source 3D printable glass pipette pullers to democratize pipette pulling.
- A platform from pipette pulling to micro-injection into the mouse brain.
- Precise and minimally invasive delivery of fluids using pulled glass pipettes into the mouse brain.
