# Supplementary figures and images for: 3D printing-based frugal manufacturing of glass pipettes for minimally invasive delivery of therapeutics to the brain
Source: Neuroprotection. Author manuscript; Available in PMC 2023 Sep 28. (PMC10538625; doi:10.1002/nep3.20)

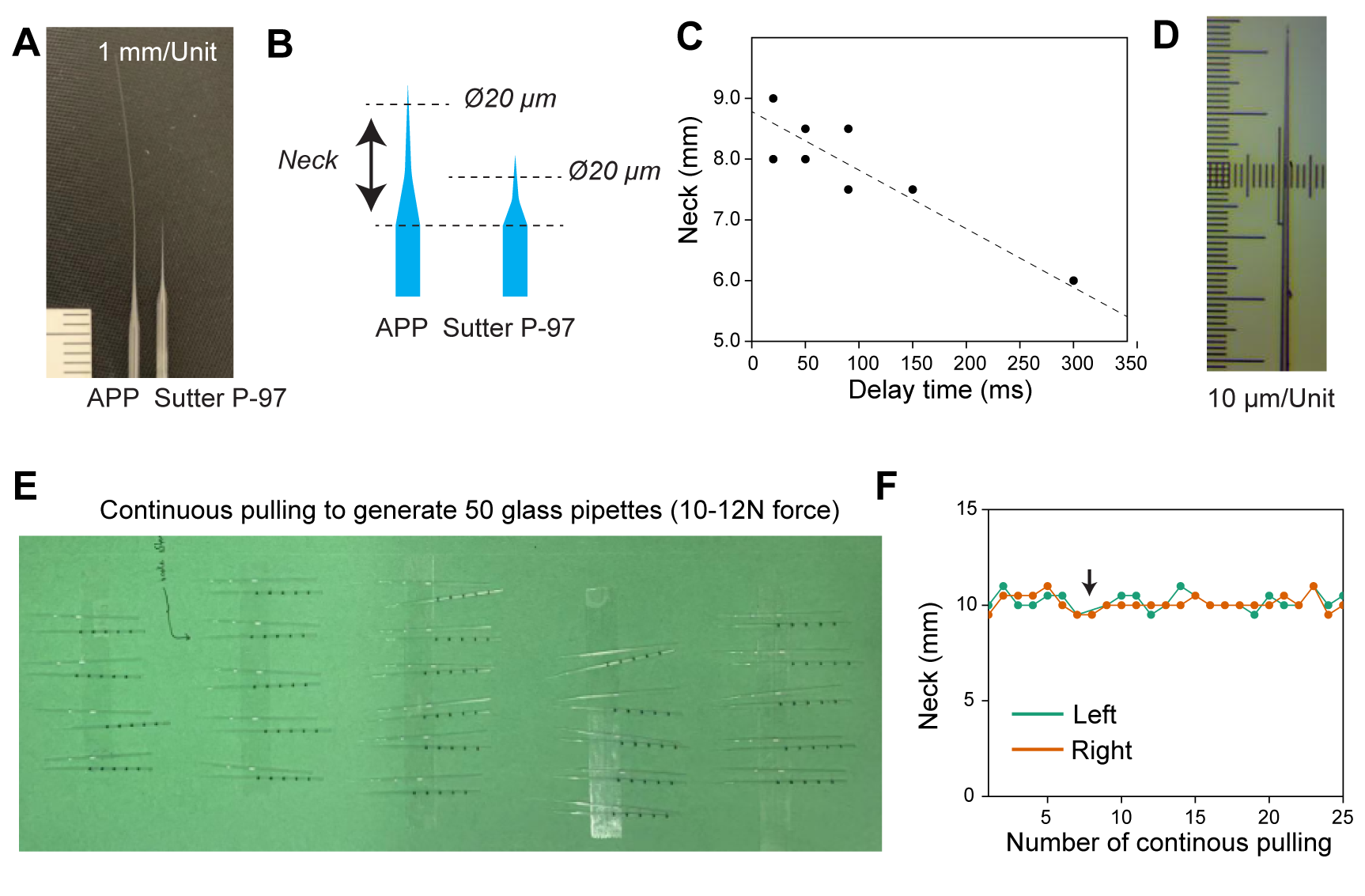

Supplement: supplementary figure [file NIHMS1930236-supplement-supplementary_figure.tif]
